# Novel *SIL1* mutations in consanguineous Pakistani families mapping to chromosomes 5q31

**Published:** 2009-05-22

**Authors:** S. Amer Riazuddin, Laleh Amiri-Kordestani, Haiba Kaul, Tariq Butt, Xiaodong Jiao, Sheikh Riazuddin, J. Fielding Hejtmancik

**Affiliations:** 1 National Centre of Excellence in Molecular Biology, University of the Punjab, Lahore, Pakistan; 2 Ophthalmic Genetics and Visual Function Branch, National Eye Institute, National Institutes of Health, Bethesda, MD

## Abstract

**Purpose:**

To investigate the genetic basis of Marinesco-Sjogren syndrome (MSS) in consanguineous Pakistani families.

**Methods:**

Two consanguineous Pakistani families with congenital cataract and muscular dystrophy were enrolled for this study. Detailed ophthalmic and systemic examination including slit lamp microscopy, electromyogram and computed tomography scans were performed to characterize the syndrome. Blood samples were collected from affected and unaffected individuals and a genome wide scan consisting of 382 polymorphic microsatellite markers was performed. Coding exons, exon-intron boundaries, 5’ UTR, and 3’ UTR of the candidate gene *SIL1* residing in the linkage interval was sequenced bi-directionally.

**Results:**

Clinical examination of the affected members of families 60067 and 60078 revealed features of MSS. The linked interval at chromosome 5q31 harbors *SIL1*. Sequencing of *SIL1* in family 60067 revealed a homozygous substitution; c1240C>T, leading to a premature substitution; p.Q414X. Similarly, sequencing of *SIL1* in family 60078 identified a homozygous change; c.274C>T, leading to a non conservative substitution; p.R92W.

**Conclusion:**

In conclusion, our data report two novel missense mutations in two consanguineous Pakistani families affected with MSS.

## Introduction

Congenital cataracts are one of the major causes of vision loss in children worldwide and are responsible for about one third of blindness in infants [1,2]. Cataract can be classified according to etiology, as environmental, hereditary, or traumatic cataract. Hereditary cataract may occur in an isolated fashion or a part of any syndrome. Marinesco–Sjogren syndrome (MSS) is a rare autosomal recessive multisystem disorder. The classical characteristics of MSS include cerebellar ataxia, short stature, mental retardation and cataracts. Additionally, skeletal abnormalities, nystagmus, hypotonia, dysarthria, and strabismus are reported in MSS patients. MSS was first described in 1931 by Marinesco in a family of Romanian origin and further characterized by Sjogren in families of Swedish origin [3].

Lagier-Tourenne and colleagues mapped the disease locus to chromosome 5q31 by homozygosity mapping in 2003 [4]. In 2005, mutations in *SIL1* were identified in individuals with MSS [5]. Further, four MSS associated loss-of-function mutations in *SIL1* leading to disturbed SIL1-HSPA5 interaction and protein folding were identified [6,7]. Additionally, since the original reports, novel mutations in *SIL1* have been identified in extended pedigrees [8,9]. However, some patients with typical MSS do not have identifiable mutations in *SIL1*, implying genetic heterogeneity [5].

SIL1 is a 461 amino-acid protein that has a potential NH_2_-terminal ER targeting sequence and a COOH-terminal tetrapeptide, most likely an ER retrieval sequence [10]. SIL1 is ubiquitously expressed and acts as an adenine nucleotide exchange factor for the heat-shock protein chaperone GRP78, a molecular chaperone functioning mainly in the endoplasmic reticulum [10,11]. SIL1 regulates the ATPase cycle of GRP78 and has thus been anticipated to be involved in protein translocation into the ER, proper folding of the newly synthesized proteins and regulating the degradation of proteins that fail to mature properly [12].

Here, we report consanguineous Pakistani families, 60067 and 60078, ascertained from the Punjab province in Pakistan. The clinical diagnosis confirmed that cataracts segregated in both families in an autosomal recessive fashion. In addition to congenital cataracts affected individuals also exhibited typical myopathic features. During the genome scan, linkage was established to chromosome 5q31 markers in both families. Sequencing of *SIL1* in family 60067 revealed a homozygous substitution, leading to a premature termination. Similarly, sequencing of *SIL1* in family 60078 identified a homozygous change that results in a non conservative substitution. These results suggest homozygous substitutions in the *SIL1* are responsible for MSS in consanguineous Pakistani families.

## Methods

### Clinical ascertainment

One hundred consanguineous Pakistani families with non-syndromic cataract were recruited to participate in a collaborative study between the National Center of Excellence in Molecular Biology, Lahore, Pakistan and the National Eye Institute, NIH, Bethesda, MD, to identify new disease loci. IRB approval was obtained for this study from the National Eye Institute and the Centre of Excellence in Molecular Biology. The participating subjects gave informed consent consistent with the tenets of the Declaration of Helsinki. The families described in this study are from the Punjab province of Pakistan. A detailed medical history was obtained by interviewing family members. Ophthalmic examinations including slit lamp microscopy were conducted to indentify the cataract phenotype. Creatine phosphokinase and aldolase kinase levels were measured through blood test performed at the Zeenat laboratory, Lahore, Pakistan. An electromyogram (EMG) was performed at the Mayo Hospital, Lahore, Pakistan to detect abnormal muscle electrical activity that can occur in many diseases and conditions, including muscular dystrophy, inflammation of muscles, pinched nerves, and peripheral nerve damage. Computed tomography (CT) scans were performed at Children Hospital, Lahore, Pakistan. The images were analyzed by doctors at the children hospital to evaluate cerebellar hypoplasia. Blood samples were collected from affected and unaffected family members. DNA was extracted by a non organic method as described by Grimberg et al. [13].

### Genotype Analysis

A genome wide scan was performed with 382 highly polymorphic fluorescent markers from the ABI PRISM Linkage Mapping Set MD-10 (Applied Biosystems, Foster City, CA) having an average spacing of 10 cM. Multiplex polymerase chain reactions (PCR) were carried out using a GeneAmp PCR System 9700 (Applied Biosystems). Briefly, each reaction was carried out in a 5 μl mixture containing 40 ng genomic DNA, various combinations of 10 μM-dye-labeled primer pairs, 0.5 μl 10X GeneAmp PCR Buffer II, 0.5 μl 10mM Gene Amp dNTP mix, 2.5 mM MgCl_2_, and 0.2 U of Taq DNA polymerase (AmpliTaq Gold Enzyme; Applied Biosystems). Initial denaturation was carried out for 5 min at 95 ºC, followed by 10 cycles of 15 s at 94 ºC, 15 s at 55 ºC and 30 s at 72 ºC and then 20 cycles of 15 s at 89 ºC, 15 s at 55 ºC and 30 s at 72 ºC. The final extension was performed for 10 min at 72 ºC and followed by a final hold at 4 ºC. PCR products from each DNA sample were pooled and mixed with a loading cocktail containing HD-400 size standards (Applied Biosystems) and loading dye. The resulting PCR products were separated in an ABI 3100 DNA analyzer and alleles were assigned using GeneScan (version 3.7; Applied Biosystems) and Genotyper Software (version 3.7; Applied Biosystems).

### Linkage Analysis

Two point linkage analyses were performed using the FASTLINK version of MLINK from the LINKAGE Program Package [14,15]. Maximum LOD scores were calculated using ILINK. Autosomal recessive cataract was analyzed as a fully penetrant trait with an affected allele frequency of 0.001. The marker order and distances between the markers were obtained from the Marshfield database and the National Center for Biotechnology Information chromosome 5 sequence maps. For the initial genome scan equal allele frequencies were assumed, while for fine mapping allele frequencies were estimated from 125 unrelated and unaffected individuals from the Punjab province of Pakistan.

### Mutation Screening

Primer pairs for individual exons were designed using the primer3 program. The sequences and annealing temperatures are available upon request. Amplifications were performed in 25 ul reactions containing 50 ng of genomic DNA, 8 picomoles each primer, 2.5 mM dNTP, 2.5 mM MgCl_2_, and 0.2 U *Taq* DNA polymerase in the standard 1X PCR buffer provided by the manufacturer (Ampli*Taq* Gold Enzyme; Applied Biosystems) PCR amplification consisted of a denaturation step at 96 °C for 5 min, followed by 40 cycles, each consisting of 96 °C for 45 s followed by 57 °C for 45 s and at 72 °C for 1 min. PCR products were analyzed on 2% agarose gel, precipitated and purified by ethanol precipitation. The PCR primers for each exon were used for bidirectional sequencing using Big Dye Terminator Ready reaction mix according to the manufacturer’s instructions (Applied Biosystems). Sequencing products were re-suspended in 10 μl of formamide (Applied Biosystems) and denatured at 95 ºC for 5 min. Sequencing was performed on an ABI PRISM 3100 Automated sequencer (Applied Biosystems). Sequencing results were assembled ABI PRISM sequencing analysis software version 3.7 and analyzed using Chromas software version 1.45.

## Results

**Figure 1:**
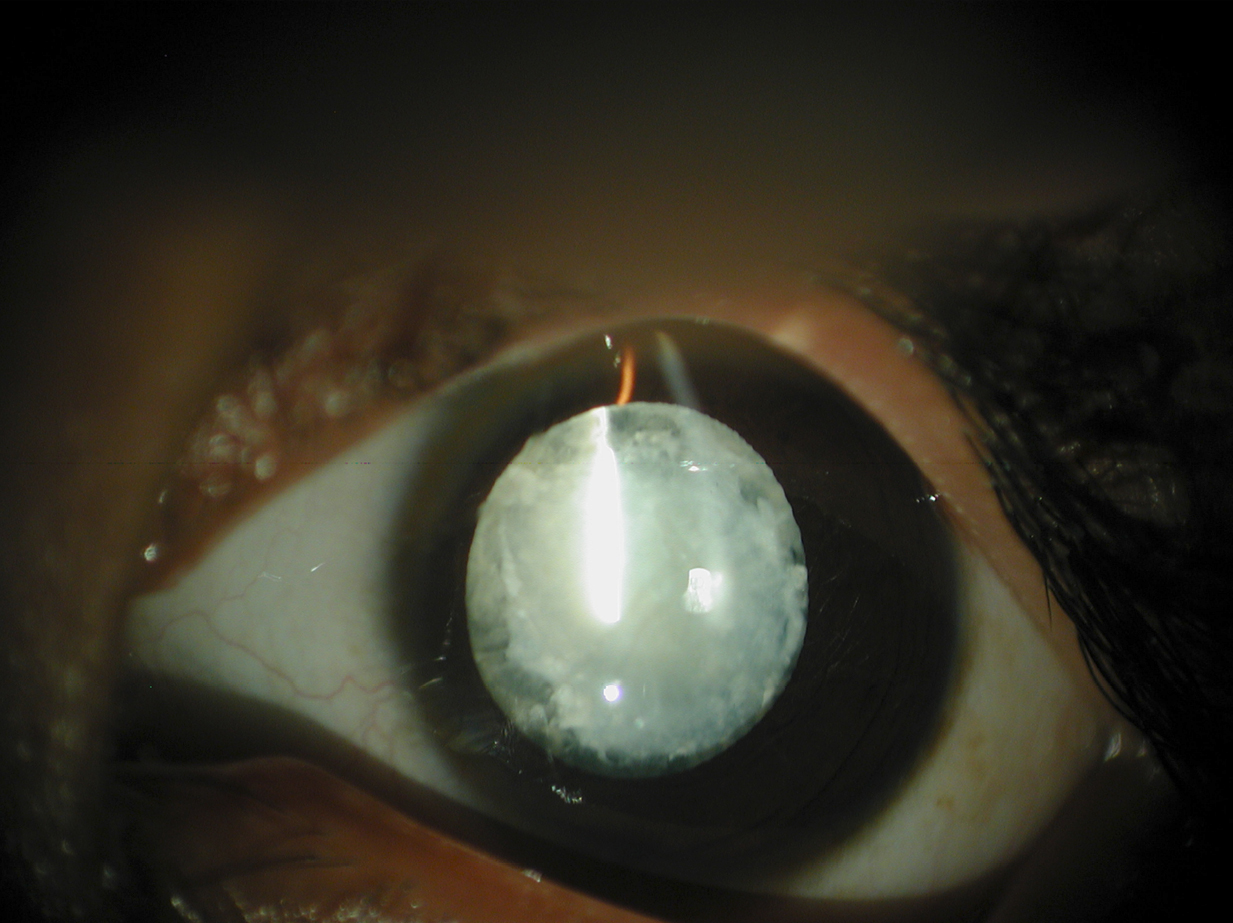
Slit lamp microscopy of bilateral membranous cataract. In family 60067 slit lamp microscopy revealed bilateral membranous cataracts in affected individual 11.

Two large consanguineous families (60067 and 60078), comprising multiple affected individuals, were recruited from the Punjab province of Pakistan. A detailed medical history was obtained by interviewing members of both families. The ophthalmic clinical records in both families revealed that cataracts in affected individuals developed in the early years of their life. In family 60067 slit lamp microscopy revealed bilateral membranous cataracts in affected individual 11 (Figure 1). The clinical records further confirmed that the rest of the affected individuals had bilateral membranous cataracts. In addition, the ophthalmic records revealed nystagmus, bilateral esotropia, and inward convergent deviation or inward deviation in the affected individuals. Skeletal abnormalities, ataxia, muscular dystrophy, mild to moderate mental retardation, dysarthria, and hypogonadism were confirmed in affected individuals of family 60067. The CK test confirmed higher than normal levels of creatine phosphokinase in affected individuals whereas aldolase levels were found to be within normal range for affected individuals (Table 1). The Electromyogram (EMG) revealed no spontaneous activity of early and/or full recruitment pattern with myogenic motor units of low amplitude and small in duration. These electrophysiological studies are indicative of non-inflammatory myopathy (muscular dystrophy).

**Table 1:** Clinical characteristics of affected individuals of family 60067.

**Clinical characteristics**	**Individual 10**	**Individual 11**	**Individual 17**	**Individual 18**	**Individual 19**
Sex	M	M	M	F	F
Age (at examination)	9 years	6 years	12 years	17 years	19 years
Cataract (Age of onset)	after 2 year	after 2year	after 2 year	after 3 year	after 3year
Cataract (phenotype)	Membranous cataract	Membranous cataract	Membranous cataract	Membranous cataract	Membranous cataract
Nystagmus	+	+	+	+	+
Ataxia	+	+	+	+	+
Mental retardation	+	+	+	+	+
Hypogonadism	NT	NT	NT	+	+
Serum CK	393.1	NT	NT	258	NT
Aldolase test	6.8	NT	NT	6.3	NT
Myopathic EMG	Muscular Dystrophy	NT	NT	Muscular dystrophy	NT
Cerebellar atrophy	NT	NT	NT	NT	NT
Dysarthria	+	+	+	+	+
Short stature	NT	NT	NT	+	+
Skeletal abnormalities	NT	NT	NT	+	+

EMG, electromyogram; CK, Creatine Phosphokinase; NT, not determined.

**Figure 2:**
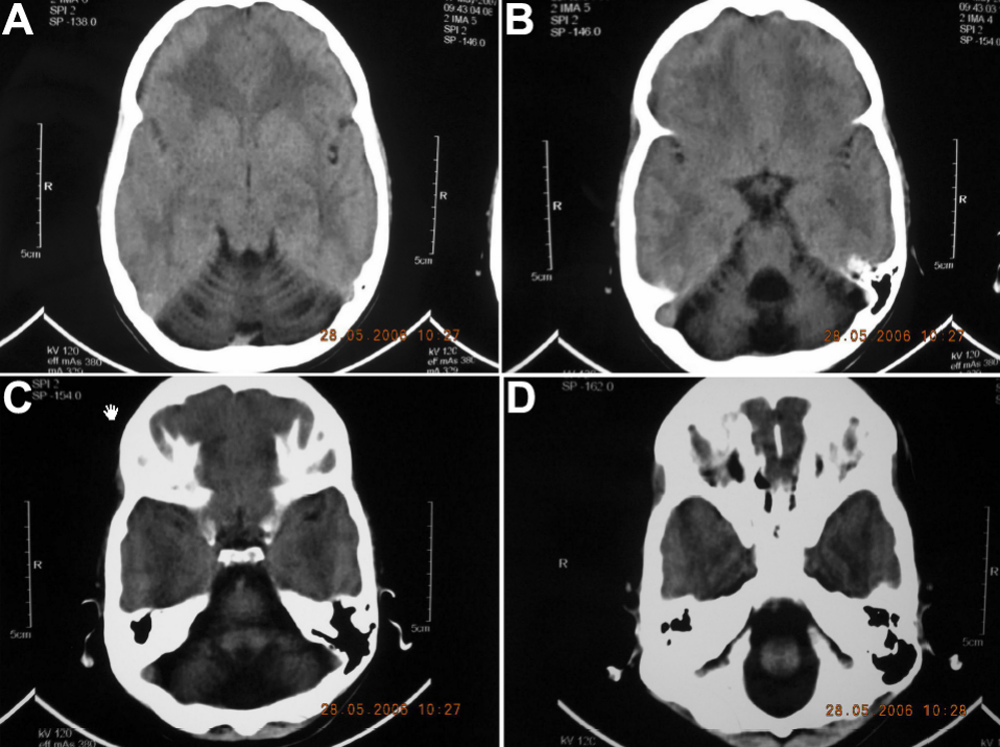
Computed tomography (CT) scan results of individual 09, family 60078. **A** depicts cerebellar dysgenesis/atrophy, reduced density of cerebellar cortex and atrophy of superior vermis, while **B** shows dilation of the 4th ventricle, lack of communication between 4th ventricle and Cisterna Magna and absence of Posterior inferior part of vermis. **C** shows the absence of inferior limit of vermis and cerebellar cortical atrophy and **D** demonstrates reduced density of the cerebellar cortex.

In family 60078 all affected individuals underwent cataract surgery during the early years of their life, hence no photograph of the cataractous lenses were available. However, the clinical report for one (individual ID 9) of the two affected individuals with slit lamp microscopy revealed cortical cataract. No information regarding the phenotype of cataract was available for the other affected individuals. All the affected individuals of 60078 can’t stand or walk without support. Further, mild mental retardation, short stature, microcephaly, and myopahty were confirmed in affected individuals of family 60078. The results confirmed higher levels of creatine phosphokinase in both affected individuals (ID 9 and 10) of 60078 whereas higher than normal aldolase levels were found in one of the two affected individuals (Table 2). The EMG studies revealed no spontaneous activity of early and/or full recruitment pattern with myogenic motor units in the tested muscles indicative of non inflammatory myopathy in both affected individuals. The results for the CT scan for both affected individuals revealed that the posterior fossa were comparatively small, dilated 4th ventricle, and extra-ventricular CSF spaces in the posterior fossa showed prominent folia and enlarged cisterna magna, whereas the third and lateral ventricles were normal without any evidence of intra/extra axial mass or hemorrhage (Figure 2). Taken together these results are indicative of cerebellar hypoplasia.

**Table 2:** Clinical characteristics of affected individuals of family 60078.

**Clinical characteristics**	**Individual 9**	**Individual 10**	**Individual 12**	**Individual 13**	**Individual 15**	**Individual 16**
Sex	M	F	M	M	M	M
Age (at examination)	13 years	8 years	12 years	14 years	15 years	17 years
Cataract (Age at diagnosis)	Congenital	Congenital	Congenital	Congenital	Congenital	Congenital
Cataract (phenotype)	Operated	Cortical cataract	Operated	Operated	Operated	Operated
Nystagmus	_	_	_	+	+	+
Ataxia	+	+	+	+	+	+
Mental retardation	+	+	+	+	+	+
Hypogonadism	NT	NT	NT	NT	NT	NT
Serum CK	516.00	270.00	NT	NT	NT	NT
Aldolase test	11.80	7.90	NT	NT	NT	NT
Myopathic EMG	Muscular dystrophy	Muscular dystrophy	NT	NT	NT	NT
Cerebellar atrophy	+	+	NT	NT	NT	NT
Dysarthria	+	+	+	+	+	+
Short stature	+	+	+	+	+	+
Skeletal abnormalities	+	+	+	+	+	+

EMG, electromyogram; CK, Creatine Phosphokinase; NT, not determined.

**Figure 3:**
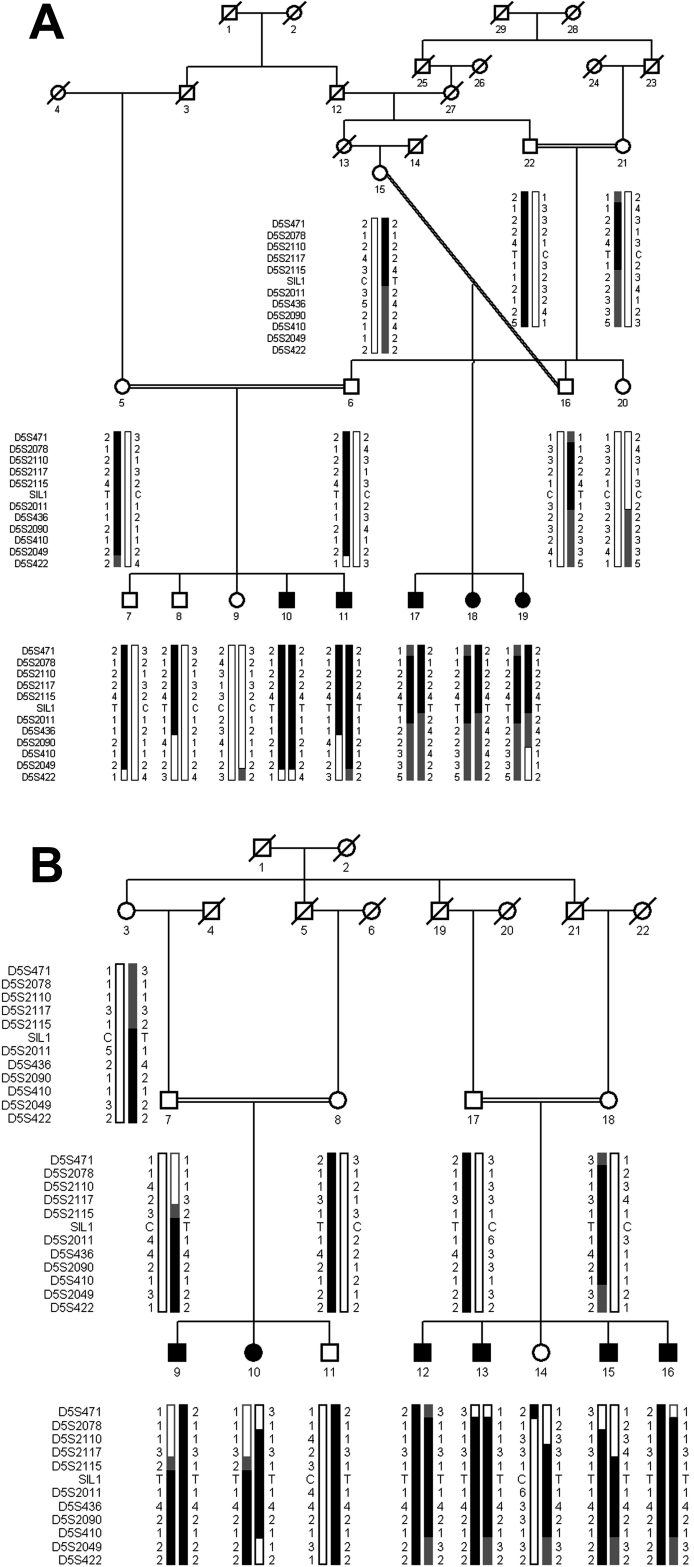
Pedigree drawings of family 60067 (**A**) and 60078 (**B**) are shown. Squares are males, circles are females, filled symbols are affected individuals, double line between individuals indicates consanguinity, and diagonal line through a symbol is deceased family member. The haplotypes of 11 adjacent chromosome 5q31 microsatellite markers are shown with alleles forming the risk haplotype are shaded black, alleles co-segregating with phenotype but not showing homozygosity are shaded grey and alleles not co-segregating with disease phenotype are shown in white.

A genome-wide scan was completed with 382 highly polymorphic STR markers. Linkage was observed with markers at chromosome 5q for both families (Table 3 and Table 4). Significant LOD scores of 3.22 and 3.08 were observed with D5S2110 and D5S2117 for family 60067 (Table 3). Similarly, significant LOD scores of 5.14, 4.02 and 3.80 were obtained with D5S2011, D5S436, and D5S2090 for family 60078 (Table 4). Visual inspection of the haplotypes supports the results and confirms linkage to chromosome 5q (Figure 3). All the affected individuals are homozygous for D5S2070, D5S2110, D5S2117, and D5S2115 in family 60067; whereas the unaffected individuals are heterozygous carriers of the disease allele or homozygous for the normal alleles. Similarly, in family 60078, all the affected individuals are homozygous for D5S2011, D5S436, D5S2090 and D5S410; whereas the unaffected individuals are heterozygous carriers of the disease allele or homozygous for the normal alleles.

**Table 3:** Two point LOD scores of markers for family 60067.

**Marker**	**cM**	**Mb**	**0**	**0.01**	**0.03**	**0.05**	**0.07**	**0.09**	**0.1**	**0.2**	**0.3**	**Zmax**	**θmax**
D5S471*	129.83	119	-2.44	0.27	0.66	0.80	0.85	0.88	0.88	0.73	0.47	0.88	0.10
D5S2078	134.72	128.19	-0.05	-0.14	-0.30	-0.46	-0.57	-0.61	-0.06	-0.21	-0.43	-0.05	0.00
D5S2110	135.25	130.8	3.22	3.15	3.00	2.85	2.71	2.56	2.48	1.71	0.95	3.22	0.00
D5S2117	137.39	133	3.08	3.02	2.90	2.78	2.65	2.53	2.46	1.82	1.17	3.08	0.00
D5S2115*	138.64	134.7	0.76	0.67	0.47	0.29	0.15	0.08	0.08	0.31	0.32	0.76	0.00
D5S2011	144.06	141.2	-4.11	-2.94	-2.61	-2.51	-2.43	-2.31	-2.22	-1.30	-0.72	-0.72	0.30
D5S436*	147.49	145.1	-4.73	-0.47	0.35	0.67	0.84	0.93	0.96	0.93	0.64	0.96	0.10
D5S2090	150.34	147.2	-∞	-1.52	-1.24	-1.19	-1.18	-1.12	-1.07	-0.44	-0.15	-0.15	0.30
D5S410*	156.47	152.7	-∞	-4.94	-4.09	-3.73	-3.43	-3.13	-2.97	-1.64	-0.87	-0.87	0.30
D5S2049	160.87	157.6	-∞	-3.01	-2.66	-2.52	-2.38	-2.19	-2.08	-1.11	-0.56	-0.56	0.30
D5S422*	164.19	162	-∞	-6.61	-4.98	-3.68	-3.25	-2.70	-2.47	-1.07	-0.42	-0.42	0.30

The asterisk indicates marker included in genome wide scan. LOD scores were calculated at different θ-values for each marker with the FASTLINK version of MLINK from the LINKAGE program package. Maximum LOD scores for each marker were calculated using ILINK.

**Table 4:** Two point LOD scores of markers for family 60078.

**Marker**	**cM**	**Mb**	**0**	**0.01**	**0.03**	**0.05**	**0.07**	**0.09**	**0.1**	**0.2**	**0.3**	**Zmax**	**θmax**
D5S471*	129.83	119	-∞	-7.86	-5.09	-3.84	-3.05	-2.48	-2.25	-0.90	-0.33	-0.33	0.30
D5S2078	134.72	128.19	-∞	-0.01	0.39	0.53	0.61	0.64	0.65	0.57	0.38	0.65	0.10
D5S2110	135.25	130.8	-∞	2.41	2.73	2.81	2.80	2.75	2.72	2.21	1.53	2.81	0.05
D5S2117	137.39	133	-∞	0.97	1.34	1.45	1.49	1.50	1.48	1.23	0.85	1.50	0.09
D5S2115*	138.64	134.7	-3.2	0.68	1.06	1.18	1.22	1.23	1.22	1.00	0.66	1.23	0.09
D5S2011	144.06	141.2	5.14	5.04	4.85	4.66	4.47	4.27	4.17	3.14	2.04	5.14	0.00
D5S436*	147.49	145.1	4.02	3.95	3.79	3.64	3.48	3.32	3.23	2.38	1.49	4.02	0.00
D5S2090	150.34	147.2	3.80	3.72	3.57	3.41	3.25	3.09	3.01	2.15	1.27	3.8	0.00
D5S410*	156.47	152.7	1.12	1.09	1.04	0.99	0.93	0.88	0.85	0.59	0.34	1.12	0.00
D5S2049	160.87	157.6	-∞	-1.05	-0.23	0.06	0.22	0.30	0.32	0.28	0.12	0.32	0.10
D5S422*	164.19	162	2.45	2.40	2.31	2.22	2.12	2.03	1.98	1.48	0.98	2.45	0.00

The asterisk indicates marker included in genome wide scan. LOD scores were calculated at different θ-values for each marker with the FASTLINK version of MLINK from the LINKAGE program package. Maximum LOD scores for each marker were calculated using ILINK.

**Figure 4:**
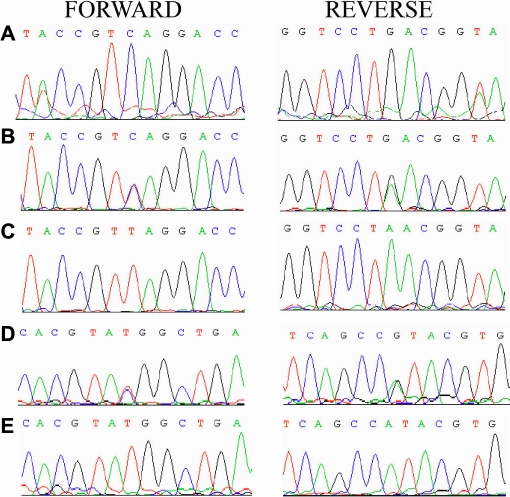
Sequence chromatograms of *SIL1*. **A**: Unaffected individual 09 in family 60067 homozygous for the wild type allele. **B**: Unaffected individual 06 in family 60067 heterozygous and **C**; affected individual 10 homozygous for the c.1240 C>T transition, leading to a premature termination p.Q414X. **D**: unaffected individual 14 of family 60078 heterozygous and **E**: affected individual 15 homozygous for the c.274 C>T transition, leading to a premature substitution; p.R92W.

*SIL1* lies on chromosome 5q31 and consists of 9 coding exons, generating a transcript of 1,895 bp that results in a 461 amino acid protein. We sequenced all coding exons, exon-intron boundaries and the 5’ and 3’ UTR regions of *SIL1*. Sequencing of *SIL1* in family 60067 revealed a homozygous substitution; c1240C>T, leading to a premature termination; p.Q414X (Figure 4A-C). All affected individuals were homozygous for the C>T transition; whereas unaffected individuals 5, 6, 7, 8, 15, 16, 21, and 22 were heterozygous for the transition. Unaffected individuals 9 and 20 are homozygous for the wild type allele. Similarly, sequencing of *SIL1* in family 60078 identified a homozygous change; c.274C>T, leading to a non conservative substitution; p.R92W (Figure 4D,E). All affected individuals were homozygous for the C>T transition; whereas unaffected individuals 3, 7, 8, 11, 14, 17, and 18 were heterozygous for the transition. These sequence variants were not found in 96 ethically matched samples from Punjab province of Pakistan.

## Discussion

Here we report two consanguineous Pakistani families characterized by autosomal recessive congenital and typical myopathic symptoms recruited from the Punjab province of Pakistan. Linkage analysis with 382 polymorphic STR markers localized the critical interval to chromosome 5q31. Sequencing of *SIL1* indentified one non-sense and a missense mutation segregating with the disease phenotype in the respective families. None of these variations were present in 96 ethnically matched samples from the Punjab province of Pakistan. Statistically significant LOD scores with chromosome 5q31 STR markers, segregation of the mutations with the disease phenotype in both families and absence in the ethnically similar control samples strongly suggests that these mutations in *SIL1* are responsible for the disease phenotype in both families.

These families were recruited to participate in a collaborative study to investigate recessive congenital cataracts. Initially, an ophthalmic evaluation was performed in Pakistan that confirmed the presence of cataracts in both families and later genome wide scans were completed jointly at the National Center of Excellence in Molecular Biology, Pakistan and the National Eye Institute, USA. After linkage was established at 5q31 and mutations were identified in *SIL1*, affected individuals in both families underwent a thorough clinical examination, which confirmed that affected individuals in both families exhibit cardinal features of MSS. One of the two mutations reported here, Q414X is present in the last exon. As there is no exon-intron boundary present downstream of the non-sense mutation, the mutant transcript is expected to not be degraded by the non-sense mediated decay, which will result in a protein that lacks 46 amino acids of its COOH-terminal. The COOH-terminus of the SIL1 protein harbors the tetrapeptide, KELR that is most likely an ER retrieval sequence. We thus hypothesize that the Q414X mutant protein is likely to lead to defective retrieval of the secretory pathway.

A majority of the reported mutations in *SIL1* are responsible for the MSS phenotype resulting from a premature termination of the protein. L457P is the only missense mutation reported that results in MSS [6]. We identified a R92W mutation segregating with the disease phenotype in family 60078. The Arg92 is conserved among higher primates and hence the substitution of a positively charged amino acid with an amino acid harboring an aromatic ring is bound to influence the protein tertiary structure and this may affect the SIL1 protein function leading to the disease phenotype in this Pakistani family.

To-date, 18 mutations have been reported in SIL1 protein that result in the MSS phenotype. This is the first report describing patients with the MSS phenotype linked with mutations in *SIL1* in families of Pakistani origin. Identification of the specific mutations in *SIL1* and the phenotype of MSS associated with these mutations will increase our understanding of the syndrome at the molecular level.
